# Health-related quality of life among patients with chronic diseases during COVID-19 pandemic: a cross-sectional study

**DOI:** 10.11604/pamj.2022.43.2.33592

**Published:** 2022-09-02

**Authors:** Ismael Ahmed, Kenenisa Tegenu, Desalew Tilahun, Samira Awel

**Affiliations:** 1 Faculty of Health Science, Institute of Health, Jimma University, School of Nursing, Jimma, Ethiopia

**Keywords:** Health-related quality of life, COVID-19 pandemic, chronic patients, Ethiopia

## Abstract

**Introduction:**

in the last two years, COVID-19 has largely changed the rhythm of human life and overwhelmed the healthcare systems globally. Patients with pre-existing chronic diseases have worse outcomes during the COVID-19 pandemic.

**Methods:**

an institution-based cross-sectional study was conducted from April 1-30, 2021. Data were collected using an interviewer-administered questionnaire and data extraction checklist. A systematic random sampling technique was used to select a total of 400 study participants. Data were entered into EPI data version 3.5.3 and exported to Statistical Package for the Social Science (SPSS) version 23.0 for analysis. Multivariable logistic regression was used and variables with a p-value < 0.05 were considered statistically significant.

**Results:**

three hundred and ten (77.5%) of the respondents had a poor overall health-related quality of life (HRQOL) during the COVID-19 pandemic. Younger age (AOR=0.10.95% CI: 0.04-0.27), no formal education (AOR=5.03, 95% CI: 1.92-13.22), shorter treatment duration(AOR=0.11, 95% CI: 0.04-0.29), presence of respiratory symptoms (AOR=9.69, 95% CI: 2.93-32.09) and missed health-care appointment during COVID-19 (AOR=3.68, 95%CI: 1.82-7.43) were significantly associated with health-related quality of life (HRQOL).

**Conclusion:**

most of the respondents had a poor overall health-related quality of life during the COVID-19 pandemic. Consideration of the influence of outbreaks on the continuity of care for a patient and focusing on contributing factors should be an essential concern of the healthcare system. The objective is to assess health-related quality and factors associated with health-related quality of life among patients with chronic diseases during the COVID-19 pandemic.

## Introduction

COVID-19 is a global pandemic with unprecedented medical, economic and social consequences that affect nations across the world [1-3]. In the past few years, the coronavirus disease (COVID-19) outbreak has significantly changed the rhythm of human life and overwhelmed the healthcare systems of many countries, including developed countries [4]. The implementation of COVID-19 measures may have raised concerns about access to treatment and quality of health care for patients with chronic diseases, and which may have affected their quality of life [5]. Pre-existing co-morbidities such as hypertension, diabetes, and cardiovascular disease are associated with the severity and higher fatality rate of COVID-19 [6]. The coronavirus disease (COVID-19) outbreak has impacted the health-care-seeking behavior of people with pre-existing chronic medical conditions which affects the quality of life of the patients. A study conducted in Singapore showed that about 40% of respondents reported missing their healthcare appointments during the COVID-19 outbreak [1]. The outbreak also has the potential to impact people with pre-existing chronic medical conditions indirectly if they are unable to access routine health care to manage their chronic conditions [1,7]. A study conducted in Turkey showed that those with greater worry about contracting COVID-19 and those who perceived themselves as very likely to die upon contracting COVID-19 were more likely to miss their health-care appointments voluntarily. This has influenced their health-care-seeking behavior for chronic medical conditions which may result in morbidity and mortality that cannot be directly attributed to the COVID-19 outbreak [1,7]. The COVID-19 pandemic had both direct and indirect effects on people with chronic diseases. This has impeded chronic disease prevention and disrupted disease management. The COVID-19 pandemic has resulted in enormous personal and societal losses, with more than half a million lives lost particularly those with chronic diseases. This impact is particularly profound [8,9]. Patients with pre-existing chronic diseases experience worse outcomes of COVID-19 infection. Beyond these direct consequences, COVID-19 has reshaped the delivery of chronic care, the non-critical cases are postponed and cancellation of chronic rehabilitation [2,10].

Routine care for chronic disease is an ongoing major challenge during a pandemic. It is important to continue routine care for people with chronic medical conditions despite the pandemic, to avoid long-term impact on their health and mortality [2,10]. Currently, most global healthcare resources are focused on coronavirus disease (COVID-19), which disrupts the continuum of care for patients with chronic diseases. A global survey showed that chronic diseases are highly impacted by a reduction in healthcare resources due to COVID-19 [11]. During a pandemic, patients and the public are under insurmountable psychological pressure which may lead to various psychological problems such as anxiety, fear, depression, and insomnia, consequently impairing health-related quality of life [6,12,13]. The study showed that the COVID-19 pandemic had a significant impact on the general population with the highest number of patients with chronic diseases presenting with stress, anxiety, and depression compared with those who did not report such diseases [6]. A study conducted in Turkey shows that 78%, 48%, 42%, and 34% of the general population felt worried, insecure, complicated, fearful, and panicked about the COVID-19 pandemic respectively. Of these 57% of them were worried, b/c of relatives who had a chronic disease were at risk of getting sick [12,14]. The World Health Organization (WHO) defines the quality of life (QoL) as individuals´ perceptions of their position in life in the context of the culture and value systems in which they live and their goals, expectations, standards, and concerns [1]. When QoL is studied under specific health conditions, it is termed health-related quality of life (HRQOL) [15]. Health-related quality of life is an essential health care indicator for diseases. It measures patients´ overall physical, mental, and emotional well-being aspects at a specific time and can be used to evaluate the severity of a disease, treatment outcomes, patient satisfaction with care, quality of services, overall patient wellbeing, and the cost-utility of interventions targeting the disease [16,17].

The ultimate goal of health care for patients with chronic diseases is not only to delay death but also to promote health and quality of life [18]. The quality of life of patients during the period of epidemic prevention and control is worse than that after epidemic prevention and control [2]. Not receiving health care (HCP) advice or guidance regularly and inconvenient treatment during the pandemic directly caused the aggravation of patients´ symptoms and a decline in their quality of life [4]. Studies show that scores of mobility; activities of daily living, emotional well-being, and bodily discomfort during the times of the pandemic were significantly higher than those before the pandemic indicating that the quality of life of patients was significantly affected during the pandemic [4,18]. Patients reported that health-related quality of life significantly deteriorated during the COVID-19 pandemic compared with before the pandemic with at least one of the EQ-5D dimensions of HRQOL during the COVID -19 pandemic with the severity of the problems in each dimension similar to or worse than pre-pandemic [5,8,19]. Consideration of the impacts of outbreaks on the continuity of care for a patient with chronic diseases in the future is an essential concern of the health system [11,12]. As part of the healthcare system of Ethiopia, continuity of care for patients with chronic diseases is affected. This would lead to an increase in the number of preventable deaths. Thus, it is important to strengthen hospital systems so that they can continue to provide care for this highly vulnerable group during the Coronavirus Disease pandemic [1]. The COVID-19 pandemic has prompted the world to explore innovative ways to continue outpatient care [13]. In developed countries, most patients with chronic diseases have follow-up through phone (telemedicine) and homes have been set up for care during pandemics, which is not true for developing countries such as Ethiopia, in which home-based care and telemedicine are unaffordable and not visible. In addition, data regarding the health-related quality of life of patients with chronic diseases during the COVID-19 pandemic have not been addressed in the present study area, thus far throughout Ethiopia. Therefore, this study aimed to assess Health-related Quality of life and factors associated to health-related Quality of life among patients with chronic diseases during the COVID-19 pandemic at the Jimma zone public hospital.

## Methods

**Study setting:** the study was conducted in public hospitals in Jimma zone, Oromia regional State, Southwest Ethiopia. The Jimma zone is 350 Km from Addis Ababa. The zone was divided into 18 districts and a town administration. There were eight hospitals (one referral and teaching hospital, one general hospital, and six primary hospitals. Of eight hospitals, study was conducted in four hospitals (Jimma University Medical Center (JUMC), Agaro Hospital, Seka Hospital, and Shanen Gibe Hospital). The population served by public health facilities is estimated to be more than 15 million annually including people from border zones and South Sudan, while the Jimma University Medical Center has a Lion share. Agaro Hospital is found in Agaro is a town to 385 Km Southwest of Addis Ababa and 45 Km from Jimma zone. In the town, there are eight clinics, two health posts, two health centers and one hospital. Shenan Gibe Hospital found in Jimma town 357 km in Southwest from Addis Ababa, capital city of Ethiopia and it provide inpatient, outpatient, emergency care, maternal and child service and chronic follow clinics. An average numbers of patient with chronic diseases attending chronic follow-up clinic of JUMC, Agaro, Seka Hospital and Shenan Gibe Hospital were 2000, 1300, 1000, and 15000 respectively.

**Study design and period:** a facility-based cross-sectional study design was conducted from April 1-30, 2021.

**Source population:** the source population for this study were all patients with chronic diseases attending chronic follow-up clinic of selected public hospitals in Jimma zone.

### Eligibility criteria

**Inclusion criteria:** adult (age ≥18-years-old) patients with at least one chronic illness on follow-up at public hospitals found in Jimma zone who visited chronic follow-up during the study period and were willing to participate in the study.

**Exclusion criteria:** severely sick patients who require urgent intervention at the time of data collection.

**Sample size determination and sampling technique:** the sample size was determined by using the single population proportion formula, considering a 50% proportion as there is no previous study conducted on this topic, 95% confidence interval level, and margin of error (5%). Finally, by considering a 10% non-response rate, the final sample size was 422.

**Sampling procedures:** there were eight public hospitals in the Jimma Zone. Of the eight hospitals, four hospitals (JUMC, Agaro Hospital, Seka Hospital, and Shanen Gibe Hospital) were selected using a simple random sampling technique. A systematic random sampling technique was used to select the study subjects by using the serial number of patients' from registration book of each hospital as a sampling frame. Participants were selected from each facility based on the number of patients with chronic disease in each facility every 13 intervals.

**Study variables, data collection tools and procedures:** validated standardized structured interviewer-administered questionnaires and patient medical records were used for data collection. Content validity of the instrument, on selected variables of interest to the research question, was examined by five experts. A Cronbach´s alpha test was done to measure the reliability of the EQ-5D-3L scale on similar populations at Limmu genet hospital on 5% of sample size and score showed high internal consistency (α= 0.86). Moreover, an English version of the questionnaire was translated to local languages (Afaan Oromo and Amharic) and reversed back to English for consistency. The questioners contain three parts, part one is sociodemographic characteristics(sex, age, educational status, marital status, religion, residence, occupation and monthly income) which were measured by tool having eight questions, part two is clinical and treatment- related characteristics (types of diagnosis, disease duration, treatment duration, taking medicines as prescribed, routine check-up during the previous year, comorbidity, source of medication, presence of respiratory symptoms in last 14 days, contact history with COVID-19 positive patients and missed health-care appointment during COVID-19) which were measured by tools having 10 questions and part 3 includes EQ-5D-3L tools to assess health-related quality of life [20]. The EQ-5D-3L includes five dimensions (mobility, self-care, usual activities, pain/discomfort, anxiety/depression), each with three levels to define possible health states (no problems, some problems, inability to/extreme problems) [21]. Each components of HRQOL has three possible score option (0, 1 and 2). A patient's response “I have no problem” on each component was scored as 0, a patient's response “I have some problems” was scored as 1, and the patient's response “I am confined to bed/severely sick” was scored as 2. Then to have the overall score of the components of HRQOL the score of each HRQOL were summed up. The outcome of interest was poor health-related quality of life defined as an accumulative score of mobility, self-care, usual activities, pain or discomfort, and depression or anxiety of six or over [2,16]. The sensitivity and specificity of the tool had been assessed in similar populations before actual data collection at Qersa hospital and its sensitivity is over 80% and the specificity is over 90% in relation to the local setting [22]. Six bachelor of science (BSc) and three master of science (MSc) nurses were recruited as data collectors and supervisors, respectively.

**Data processing and analysis:** both data collectors and supervisors were trained for three days on the objectives of the study and data collection techniques. A supervisor checked the completeness and consistency of the questionnaire. The principal investigator evaluated data before analysis to verify the completeness of the collected data. A pretest was performed on 5% of the sample size at Limmu Hospital to ensure internal consistency of the instrument. The data were entered and coded into Epi-Data version 3.5.3, and exported to SPSS version 23.0 for analysis. The mean and standard of deviation of the components of HRQOL were computed. Variables with a p-value <0.25 on bivariate logistic regression were candidate for multivariable logistic regression analysis and Variables with a p-value < 0.05 on multivariable logistic regression were considered statistically significant. In this study we did not have any missing values in the data set.

**Ethical approval and consent:** ethical clearance was obtained from the Institutional Review Board of Jimma University, Institute of health. A formal letter from the Institute of Health Science was submitted to selected public hospitals to acquire their co-operation. Ethical issues within the study were taken into consideration when carrying out the study. At the initial stage of data collection, informed oral and written consent was taken from respondents and the participants were assured that their participation was recorded anonymously. Participants were informed of the purpose, merit, and demerits of the study, and their participation is voluntary. The obtained data was used only for research purposes and kept confidentially.

## Results

**Sociodemographic characteristics:** of the calculated 422 samples, a total of 400 study participants were included in the study. From a total of the study participants, 230 (57.5%) of them were female. Most of the study participants 178(44.5%) were in the age category of ≧50 years. About 158(39.5%) of respondents had no formal education (Table 1).

**Table 1:** socio-demographic characteristics of study participants attending follow-up during the COVID-19 pandemic at public hospitals in Jimma zone, South-West Ethiopia, 2021 (n=400)

Variables	Categories	Frequency (No)	Percent (%)
**Sex**	Male	170	42.5
	Female	230	57.5
**Age in year**	≤28	34	8.5
	29-39	68	17.0
	40-50	120	30.0
	>50	178	44.5
**Marital status**	Single	48	12.0
	Married	318	79.5
	Widowed	32	8.0
	Divorced	2	0.5
**Religion**	Muslim	214	53.5
	Orthodox	148	37.0
	Protestant	38	9.5
**Educational status**	No formal education	158	39.5
	Primary school	140	35.0
	Secondary education	58	14.5
	College and above	44	11.0
**Residence**	Urban	212	53.0
	Rural	188	47.0
**Occupation**	Government employee	82	20.5
	Merchant	56	14.0
	Farmer	144	36.0
	House wife	80	20.0
	Other	38	9.5
**Monthly income in ETB**	>1000	98	24.5
	≤1000	302	75.5

**Clinical and treatment characteristics of study participants:** hypertension was the most common type of diagnosis among chronic follow-ups at public hospitals in the Jimma zone, which accounts for 122 (30.5%). About 80 (20%) of the respondents had experienced respiratory symptoms in the last 14 days. Forty six percent (11.5%) of respondents had a contact history with COVID-19 positive confirmed patients. More than one-third, 154 (38.5%) of the respondents have missed their healthcare appointments during the COVID 19-pandemic (Table 2).

**Table 2:** clinical and treatment characteristics of study participants attending follow-up during the COVID-19 pandemic at public hospitals in Jimma zone, South-West Ethiopia, 2021 (n=400)

Variables	Categories	Frequency	Percent
Types of diagnosis	Diabetes mellitus	110	27.5
	Hypertension	122	30.5
	CHF or other cardiovascular diseases	76	19.0
	Neurologic disorders	36	9.0
	Asthma/Chronic obstructive pulmonary disease	14	3.5
	Renal failure / other renal disease	4	1.0
	Liver failures	6	1.5
	Other	32	8.0
Disease duration in years	≤5	222	55.5
	6-10	58	14.5
	>10	120	30.0
Treatment duration in years	≤5	236	59.0
	6-10	91	22.8
	>10	73	18.2
Do you take your medicines as prescribed	Yes	372	93.0
	No	28	7.0
Routine check-up during the Previous year	Yes	342	85.5
	No	58	14.5
Presence of comorbid diseases	Yes	140	35.0
	No	260	65.0
Source of medication	Free	116	29.0
	Payments	284	71.0
Presence of respiratory symptoms in last 14 days	Yes	80	20.0
	No	320	80.0
Contact history with COVID-19	Yes	46	11.5
	No	354	88.5
Missed health-care appointment during COVID 19	Yes	154	38.5
	No	246	61.5

**Components of HRQOL, overall health-related quality of life and factor associated with HRQOL among patients with chronic diseases:** the mean scores and standard deviations of the five dimensions of the EQ-5D: mobility, self-care, usual activities, pain/discomfort, and anxiety/ depression, were 1.61 ± 0.632, 1.55, ±0.655, 1.61 ±0.707, 1.60 ± 0.592, and 1.65±0.707 respectively. The most frequently reported problem was pain/discomfort followed by mobility. More than one-third of 146 (36.5%) of the respondents have had some problems with the self-care components of HRQOL. One forty (35.0%) of respondents have had some limitations with performing usual activities. About 310 (77.5%) of the respondents had poor overall health-related quality of life (HRQOL) during the COVID-19 pandemic (Table 3). On binary logistic regression; age, educational status, residence, occupation, monthly income, disease duration, treatment duration, taking medicines as prescribed, routine check-ups during the previous year, presence of comorbid diseases, presence of respiratory symptoms in the last 14 days, contact history with COVID-19 and missed healthcare appointment during COVID-19 were variables candidate for multivariate logistic analysis with a p-value <0.25. During the multivariate logistic analysis age, educational status, treatment duration, presence of respiratory symptoms in the last 14 days, and missed healthcare appointments during COVID-19 were significantly associated with health-related quality of life. Respondents in age categories of ≦ 28 years old (AOR =0.10, 95% CI: 0.04- 0.27, P=0.001) were 90.0% time less likely to have a poor health-related quality of life compared to those in the age group of >50 years old. Patients who had no formal education were 5 times (AOR=5.03, 95% CI: 1.92-13.22, P=0.001) more likely to have a poor overall health-related quality of life compared to those who had completed college and above. Respondents who were on treatment for ≥5 years (AOR =0.37, 95% CI: 0.15- 0.92, P=0.030), 6-10 years (AOR=0.11, 95% CI: 0.04- 0.29, P=0.001) were 63.0% and 89.0% time less likely to have poor health-related quality of life compared to those who were on treatment for >10 years respectively. Respondents who had manifested respiratory symptoms in their last 14 days were 9.7 times ((AOR=9.69, 95% CI: 2.93-32.09, P=0.001) more likely to have poor overall health-related quality of life compared to those who had not manifested respiratory symptoms in their last 14 days. Patients who missed healthcare appointments during the COVID-19 pandemic were 3.7 times (AOR=3.68, 95% CI: 1.82-7.43, P=0.001) more likely to have poor overall health-related quality of life compared to those who did not miss health-care appointments during the COVID-19 pandemic (Table 4).

**Table 3:** components of HRQOL of patients attending follow-up during the COVID-19 pandemic at public hospitals in Jimma zone, South-West Ethiopia, 2021 (n=400)

Dimensions of HRQOL	Categories	Frequency (No)	Percent (%)
Mobility	I have no problem in walking about	180	45.0
	I have some problems in walking	188	47.0
	I am confined to bed	32	8.0
self-care	I have no problem with self-care	218	54.5
	I have some problems washing or dressing my self	146	36.5
	I am unable to wash or dress my self	36	9.0
Usual activities	I have no problem with performing my usual activities	208	52.0
	I have some with performing my usual activities	140	35.0
	I am unable to perform my usual activities	52	13.0
Pain/discomfort	I have no pain or discomfort	182	45.5
	I have moderate pain or discomfort	196	49.0
	I have extreme pain or discomfort	22	5.5
Anxiety/depression	I am not anxious or depressed	196	49.0
	I am moderately anxious or depressed	150	37.5
	I am extremely anxious or depressed	54	13.5

**Table 4:** factors associated with self-management practice among patients with chronic disease on follow-up at public hospitals in Jimma zone, South-West Ethiopia, 2021 (n=400)

Variables	Category	Overall HRQOL		COR (95% CI)	AOR (95% CI)	p-value
		Poor HRQOL	Less likely poor HRQOL			
Age in year	<=28	14 (41.2)	20 (58.8)	0.19(0.09-0.41)	0.10(0.04- 0.27)	0.001*
	29-39	561 (82.4)	12 (17.6)	1.27 (0.62-2.60)	0.91(0.39-2.11)	0.820
	40-50	100 (83.3)	20 (16.7)	1.36 (0.75-2.47)	1.09 (0.55-2.16)	0.811
	>50	140 (78.7)	38 (21.3)	1	1	
Educational status	No formal education	140 (88.6)	18 (11.4)	3.63 (1.63-8.09)	5.03 (1.92-13.22)	0.001*
	Primary school	100 (71.4)	40 (28.6)	1.17 (1.56- 2.43)	1.59 (0.67-3.82)	0.290
	Secondary education	40 (69.0)	18 (31)	1.04 (0.45- 2.41)	1.38 (0.49-3.83)	0.541
	College and above	30(68.2)	14 (31.8)	1	1	
Residence	Urban	152(71.7)	60 (28.3)	0.48 (0.29- 0.79)	1.29 (0.64-2.59)	0.470
	Rural	158 (84)	30 (16)	1	1	
Occupation	Government employee	54 (65.9)	28 (34.1)	1.13 (0.51-2.51)	0.67 (0.24-1.88)	0.440
	Merchant	40 (71.4)	16 (28.6)	1.46 (0.61-3.51)	0.90 (0.30-2.71)	0.850
	Farmer	132 (91.7)	12 (8.3)	6.42 (2.65-15.55)	2.19 (0.72-6.64)	0.172
	House wife	60 (75)	20 (25)	1.76 (0.76-4.02)	1.16 (0.39-3.42)	0.780
	Other	24(63.7)	14(36.8)	1	1	
Monthly income in ETB	< 1000	82 (83.7)	16 (16.3)	1.66 (0.92- 3.02)	2.16 (0.99-4.74)	0.054
	≥1000	228 (75.5)	74 (24.5)	1	1	
Disease duration in years	≤5	180 (81.9)	42 (18.9)	0.86 (0.48-0.54)	1.42 (0.53-3.80)	0.480
	6-10	30 (51.7)	28 (48.)3	0.21 (0.11- 0.43)	0.70 (0.24-2.06)	0.521
	>10	100 (83.3)	20 (16.7)	1		
Treatment duration in years	≤5	185 (78.4)	51 (21.6)	0.45 (0.20-0.99)	0.37 (0.15-0.92)	0.030*
	6-10	60 (65.9)	31 (34.1)	0.24 (0.10-0.56)	0.11 (0.04-0.29)	0.001*
	>10	65(89.0)	8(11.0)	1		
Do you take your medicines as prescribed	Yes	284 (76.3)	88(23.7)	0.25 (0.06-1.07)	1.85 (0.31-10.92)	0.493
	No	26 (92.9)	2 (7.1)	1	1	
Routine check-up during the previous year	Yes	256 (74.9)	86 (25.1)	0.22 (0.08-0.63)	0.48 (0.14-1.59)	0.230
	No	54 (93.1)	4 (6.9)	10	1	
Presence of comorbid diseases	Yes	114 (81.4)	26 (18.6)	1.43 (1.86- 2.39)	1.37 (0.68-2.73)	0.381
	No	196 (75.4)	64(24.6)	1	1	
Presence of respiratory symptoms in last 14 days	Yes	76 (95)	4 (5)	6.98 (0.05-0.40)	9.69 (2.93- 32.09)	0.001*
	No	234 (73.1)	86 (26.9)	1	1	
Contact history with COVID-19	Yes	44(95.7)	2 (4.3)	7.28(1.73-13.64)	4.20 (0.95-18.70)	0.059
	No	266 (75.1)	88 (24.9)	1	1	
Missed health-care appointment due to COVID 19 pandemic	Yes	140(90.9)	14(9.1)	4.4(2.42- 8.25)	3.68(1.82-7.43)	0.001*
	No	17(69.1)	76 (30.9)	1	1	

## Discussion

The present study shows that 310 (77.5%) of the patients attending chronic follow-up at public hospitals in the Jimma zone had a poor overall health-related quality of life (HRQOL) during the COVID-19 pandemic. Age, educational status, duration of treatment, presence of respiratory symptoms, and missed healthcare appointments during COVID-19 were significant variables associated with HRQOL. The finding of this study is supported by studies conducted in India, Turkey, Asia, Morocco, and Ethiopia which reported that these variables were significantly associated with health-related quality of life of patients [12,14,15,19]. This study identified that, the younger patients were less likely to have poor HRQOL during COVID-19 pandemic compared with elder patient, which is supported by other studies conducted in Ethiopia and Morocco [2,19] which were reported that younger patients were less likely had poor HRQOL compared with elder patient. This might be because physical and psychological functioning diminishes with age, leading to comorbidities and the onset of complications which contribute to affected health-related quality of life of patients. Patients who had no formal education had poor health-related quality of life compared to those who completed college and above.

The finding of this study is supported by other studies which report that as levels of education increase, the likelihood to have affected health-related quality is decreased [15]. The justification might be that those who, more educated might be more aware of health and practice better prevention strategies and healthy lifestyles in their lives. Respondents who were on treatment for <= 5 years and 6-10 years were likely to have poor health-related quality of life compared to those who were on treatment for >10 years respectively. The finding of this study is supported by the study conducted in South Central Ethiopia [1]. This might be related to the adherence level of the patients to medication which may relate to the duration of treatment. Patients who had respiratory symptoms in the last 14 days were more likely to have a poor health-related quality of life compared to those who did not have respiratory symptoms in the last 14 days. This might be related to an assumption in the community concerning symptoms of COVID-19. The community perceived that most of the patients having respiratory symptoms are more likely to have COVID-19. So that those who have respiratory symptoms consider themselves, as they are COVID-19 positive, which has significance on the impact of the psychology of the patients which contributes to a decrement of their overall health-related quality of life. Not access to receive HCP advice or guidance regularly and inconvenient treatment during the pandemic directly caused the aggravation of patients´ symptoms and the decline in their quality of life. In this particular study, respondents who missed healthcare appointments during the COVID-19 pandemic had a poor health-related quality of life compared to those who did not miss their healthcare appointment.

**Limitations of this study:** this particular study was limited to hospitals founds in Jimma zone, which might not be generalized to the total population across the countries. Using face-to-face interviews may lead to social desirability bias by overestimating or underestimating the result. This study was conducted using a cross-sectional study design in which the link between the outcome and the exposure cannot be determined because of the exposure and outcome were simultaneously assessed.

**Funding:** the study was funded by Jimma University, Ethiopia and the funder had no interference with the conduction, analysis, and publication process.

## Conclusion

Even though the health-related quality of life (HRQOL) is an essential health care indicator for diseases, this study found that a high number of patients attending chronic follow-up at public hospitals in the Jimma zone has affected the overall health-related quality of life (HRQOL) during the COVID-19 pandemic which highlights the prevalent problematic degree of HRQOL of patients. Age, educational status, treatment duration, presence of respiratory symptoms within the last 14 days, and missed health-care appointments during COVID-19 were significantly associated with health-related quality of life. Therefore, clinical follow-up and psychological treatment should be encouraged for these groups, and it is important to strengthen hospital systems so that they continue to provide care for this highly vulnerable group during the Coronavirus Disease-19 pandemic. The consideration of the impact of the outbreaks on the continuity of care for a patient with chronic diseases in the future should be an essential concern of the health system.

### What is known about this topic

- *COVID-19 is a global pandemic with unprecedented medical, economic and social consequences that affect nations across the world;*
- *Globally, different measurement is undertaken to tackle the increased spread of the COVID-19;*
- *Patient with chronic diseases are at higher risk of COVID-19 viral infection, with great morbidity and mortality*.

### What this study adds

- *High proportion of patient with chronic diseases had poor overall health-related quality of life during the COVID-19 pandemic;*
- *Younger age, no formal education, short treatment duration, presence of respiratory symptoms in the last 14 days and missed healthcare appointment during COVID-19 were variables significantly associated with health-related quality of life;*
- *The findings will be used to design interventions; to avoid and overcome barriers of health-related quality of life of patients with the chronic disease during the COVID-19*.
